# Brief Online Training Enhances Competitive Performance: Findings of the BBC Lab UK Psychological Skills Intervention Study

**DOI:** 10.3389/fpsyg.2016.00413

**Published:** 2016-03-30

**Authors:** Andrew M. Lane, Peter Totterdell, Ian MacDonald, Tracey J. Devonport, Andrew P. Friesen, Christopher J. Beedie, Damian Stanley, Alan Nevill

**Affiliations:** ^1^Institute of Sport, University of WolverhamptonWalsall, UK; ^2^Department of Psychology, University of SheffieldSheffield, UK; ^3^Department of Sport and Exercise Sciences, Canterbury Christ Church UniversityCanterbury, UK; ^4^Faculty of Health and Life Sciences, Coventry UniversityCoventry, UK

**Keywords:** intervention, emotion, mood, self-regulation, performance

## Abstract

In conjunction with BBC Lab UK, the present study developed 12 brief psychological skill interventions for online delivery. A protocol was designed that captured data via self-report measures, used video recordings to deliver interventions, involved a competitive concentration task against an individually matched computer opponent, and provided feedback on the effects of the interventions. Three psychological skills were used; imagery, self-talk, and if-then planning, with each skill directed to one of four different foci: outcome goal, process goal, instruction, or arousal-control. This resulted in 12 different intervention participant groups (randomly assigned) with a 13th group acting as a control. Participants (*n* = 44,742) completed a competitive task four times—practice, baseline, following an intervention, and again after repeating the intervention. Results revealed performance improved following practice with incremental effects for imagery-outcome, imagery-process, and self-talk-outcome and self-talk-process over the control group, with the same interventions increasing the intensity of effort invested, arousal and pleasant emotion. Arousal-control interventions associated with pleasant emotions, low arousal, and low effort invested in performance. Instructional interventions were not effective. Results offer support for the utility of online interventions in teaching psychological skills and suggest brief interventions that focus on increasing motivation, increased arousal, effort invested, and pleasant emotions were the most effective.

## Introduction

Many tasks are competitive, whether these are job interviews, driving tests, school examinations, business deals, or sport competitions. During tasks where individuals place high personal importance on goal achievement, they may also experience intense emotions, which in turn can have powerful effects on thoughts and actions (Lazarus, 2000; Baumeister et al., 2007; Lane et al., 2012). Psychological skills that help people regulate emotions and cope with the demands of competition, delivered through effective interventions, would have large appeal.

With an estimated 3 billion internet users worldwide, interventions delivered online could have considerable utility. Recent research has emphasized the potential of online interventions in different areas of application including behavioral change (Webb et al., 2010; Brouwer et al., 2011; Kohl et al., 2013), health (Cugelman et al., 2011), and clinical practice (Gaffney et al., 2013). A recent review of the effectiveness of interventions argued that future research is needed to not only test the efficacy of such interventions but also examine the active parts of training (Webb et al., 2010).

Psychological skills comprise learned behaviors that with appropriate practice can enhance performance, increase enjoyment, or achieve greater satisfaction of competition (Kremer and Moran, 2008). Of the plethora of psychological skills reported (Thomas et al., 1999; Thelwell, 2015), self-talk (a person's inner dialogue), and imagery (using images for situation rehearsal) are frequently used and effective (Guillot and Collet, 2008; Hatzigeorgiadis et al., 2011, 2014; Tod et al., 2011; Macintyre et al., 2013). Implementation intentions (Gollwitzer and Sheeran, 2006) present a psychological skill commonly used in health-behavior change contexts that is increasingly used in competitive contexts (Achtziger et al., 2008). Implementation intentions take the form of if-then plans, e.g., “*If* I start to doubt myself…*then* I will remind myself I have the skills!” The “*if”* identifies a specific goal-pertinent situational cue (i.e., a good opportunity to act, or a moment of self-doubt), whilst the *then* identifies a specific planned response that should help achieve the intended goal. Framing goal intentions in the form of an if-then plan has been found to be more effective than goal intentions because the “*then”* aspect (usually a goal-related response) is automatically primed by the presentation of the opportunity to overcome a barrier to performance, identified in the “*if”* part (Achtziger et al., 2008).

Psychological skills can be focused on increasing motivation via use of outcome goals (e.g., “placing first”) and process goals (e.g., “I can try to react quicker”). Psychological skills can also be focused on technical instructions (e.g., “concentrate on the ball”) or arousal control (e.g., reducing or increasing arousal intensity). Research has found that the type and focus of the psychological skill influences its effectiveness in the context where it is used. Theodorakis et al. (2000) argued that instructional self-talk (technical and tactical focus) could be helpful in tasks requiring fine motor skills, where information on precision of movement rather than speed or strength of movement determines success. Conversely, motivational self-talk (outcome and process focus) is considered more appropriate for tasks characterized by strength and endurance (Blanchfield et al., 2014). Psychological skills can also include strategies to reduce or increase the intensity of the physiological and cognitive aspects of emotion as directly as possible (Gross and Thompson, 2007; Hanin, 2010). Arousal-control interventions include progressive muscular relaxation, some forms of mindfulness, and listening to music. Although relaxation techniques are commonly used in practice, the volume of research to investigate their effects in competition is minimal (Kudlackova et al., 2013). A study that compares and contrasts the effects of different psychological skills with a different focus would cast light on the contextual applicability of them. To date, no such study has been published. Given the reach of online interventions and the value in teaching people psychological skills to help perform better in competitive situations, the aim of the present study was to test the efficacy of different psychological skills designed to help people perform better in competition through an online intervention platform.

In the present study, we worked with the British Broadcast Corporation (BBC) Lab UK to develop and test the effects of an online intervention program. We designed an online task that incorporated inter-personal competition in order to create pressure conditions so that participants would seek to regulate emotions as part of mental preparation (Lane et al., 2012). Evidence shows that emotions are predictive of performance across different areas of application (Lazarus, 2000; Baumeister et al., 2007; Hanin, 2010; Lane et al., 2012).

Emotions usually encompass three types of response: (a) cognitive such as changes in attention, perception, and information processing priorities, (b) physiological, such as changes in respiration and heart rate, and (c) behavioral, such as increasing the intensity of actions (Lane et al., 2012). Emotions function as a signal to the individual of the congruence between the prevailing situational challenges and the individual's immediate priorities (Lazarus, 2000). Emotions could aid performance via their associated “action tendency” that is, a hard-wired predisposition to act in accordance with the cause of the emotion. Thus, emotions might catalyze processes that provide extra energy to help the individual increase effort in an attempt to perform better (Lazarus, 2000). Emotion regulation in the context of performing under pressure involves strategies to alter perceptions of the task, or the intensity of emotions directly via arousal control (Gross and John, 2003; Gross and Thompson, 2007; Lane et al., 2012).

The performance task was a simple concentration grid which is a task that has been found to be related to variations in emotions (Lane et al., 2004). The task was designed to be competitive as it involved inter-personal competition. In the performance task in the present study, this involved competing against a computer generated opponent that was matched against the previous performance of the participant. Therefore, we investigated the effects of psychological skills on performance under pressure, and changes in arousal, emotion, and effort invested. We investigated three different skills: imagery, self-talk, and if-then planning. Each skill had variations focused on one of four different foci: outcome goal, process goal, instructional, or arousal-control. In a meta-analysis of emotion regulation strategies, Webb et al. (2012) found that reappraisal was the most effective emotion regulation strategy. Interventions focused on motivational and instructional processes should lead to an increased belief in ability to perform the task successfully, and consequently, emotion regulation via re-appraisal. Webb et al. (2012) found that strategies that try to regulate the physiological arousal associated with the emotion itself were less effective.

The effects of regulating physiological arousal will vary substantially as arousal-performance relationships vary between activities. For example, in the broad context of sport, low arousal is associated with better performance in sports such as archery and high arousal is associated with better performance in sports such as power-lifting. In a within-subject study, Lane et al. (2009) found people preferred to experience higher anger for sport in contrast to academic examination. Hanin (2010) found that the intensity through which an emotion will be helpful or unhelpful is highly individualized. Hence, some people will seek to reduce the intensity of unpleasant emotions and others will seek to increase them depending on how close they are to their optimal state (Hanin, 2010; Lane et al., 2011, 2012). The optimal state for some individuals is to feel intense arousal and unpleasant emotions, and low arousal and pleasant emotions in others. However, whilst regulation of arousal could be focused on increasing or reducing the intensity, a recent study reported athletes rarely used strategies to increase the intensity of unpleasant emotions, but to regulate it to an optimum (Lane et al., 2015). Lane et al. (2015) suggested that the greater challenge is to reduce the intensity of unpleasant emotions to prevent poor performance through over-arousal.

The key question is which psychological skill associated with the greatest improvements? First, we hypothesized that performance would improve, partly through physical practice (Stafford and Dewar, 2014), that is, performance would improve in each group including the control group. Second, we hypothesized that each intervention would lead to better performance in comparison to the control group. We compared and contrasted the effects of the intervention foci in terms of their effects on emotions, arousal and effort. Motivational interventions should lead to improved performance via an increased intensity of pleasant emotion, arousal and effort (Lane et al., 2004; Blanchfield et al., 2014). Instructional interventions should lead to improved performance via making fewer errors (Theodorakis et al., 2000), and whilst emotions might be functional, arousal and effort are less likely to be increased. Interventions that promote individuals to focus on arousal-control (Gross and Thompson, 2007) should reduce the intensity of unpleasant emotions such as anxiety, and as high anxiety is associated with poor attentional control, improved performance could be explained by making fewer errors (Easterbrook, 1959).

As these interventions were delivered through three psychological skill techniques, we could explore which technique was most effective. If-then strategies that require little rehearsal could produce more rapid performance improvements than strategies such as arousal-control, self-talk and particularly imagery, which require more rehearsal to become automated (Guillot and Collet, 2008; Tod et al., 2011; Macintyre et al., 2013).

Given online delivery of interventions is novel in this context, we assessed participant's perceptions of their perceived effectiveness in terms of whether following an interventions helped regulate emotions and improve performance. Meta-emotional beliefs are knowledge that a person has about her or his previous experiences (Hanin, 2010). Hanin (2010) found that meta-emotional beliefs have a powerful influence on regulation behaviors. When an individual associates goal attainment with specific emotions, this increases the likelihood of those emotions re-occurring in similar situations in the future. It is argued that their meta-beliefs will be modified in the light of subsequent experiences. Therefore, if people believed the intervention followed helped them perform better, and that they were in the appropriate emotional state to perform, then this increases the likelihood they will be more likely to use the intervention in the future. Although the present study was delimited to using the intervention in one setting, examination of the effects of interventions using objective performance measures, subjective performance measures and beliefs in their effectiveness offers helpful information to guide future research and practice.

## Materials and methods

### Participants

Participants were 44,742 volunteers recruited to the project via the BBC Lab UK website (Age 16–91 years; M = 34.81, SD = 13.84; Male, *n* = 27,945, Female *n* = 16,797). Of these, 17,038 (38.1%) had an educational degree or professional qualification, 8949 (20%) had a postgraduate degree and 6052 (13.5%) were still in education. In terms of employment, 20,939 (48.8%) were in full-time employment; 3065 (6.9%) were in part-time employment; 4704 (10.5%) were at school; 7323 (16.4%) were at university; 4041 (9%) were self-employed; 768 (1.7%) were at home or a full-time parent; 1872 (4.2%) were unemployed; and 2030 (4.5%) had retired. To qualify for the sample, participants were required to: (a) not have taken the test before, (b) have audio facilities on their computer or device, (c) use a mouse rather than a keyboard to complete the game, (d) complete all rounds of the game, and (e) have received both “doses” of the online video intervention.

### Procedure

Ethical approval was granted by the first author's university ethics board. BBC Lab UK launched a campaign to recruit participants via a promotional film and news features on national television and radio programs. Data were collected online via the BBC Lab UK website (see https://www.bbc.co.uk/labuk/). Participants received one of three psychological skill interventions (self-talk, imagery, or if-then planning) directed at one of four different aspects of performance (process, outcome, arousal-control, or instruction) resulting in 12 different participant groups. A 13th control group received instructions that encouraged reflection on performance, and was neutral in terms of giving instructions or raising motivation (see Table 1).

**Table 1:** **Intervention and examples of intervention scripts**.

**Psychological skill:**	**Focus of intervention**			
	**Process**	**Outcome**	**Arousal-control**	**Instruction**
Self-talk	“I can react quicker this time”	“I can beat my best score”	“I will stay calm”	“I will focus completely on each number I need to find”
Imagery	“I want you to picture yourself playing the game, knowing that you can react quicker than you did last time.”	“I want to you to picture yourself playing the game, and imagine beating your best score.”	“I want to you to picture yourself sat in front of the computer calmly with no tension in your body.”	“See yourself scanning the whole grid to find the next number, moving the pointer back to middle of grid after finding each one.”
If-then planning	“IF I start worrying about mistakes, THEN…I will say to myself, “Good performance last time. I can do it again!”'	“IF I can't find the number THEN…I will tell myself that I can beat my best score!”	“IF I find myself holding my breath, THEN …I will say to myself, ‘I will stay focussed!’“	”IF I lose my concentration, THEN …I will move the pointer to the middle of the grid after each number!”
Control	“You have played the game now. You have to find the numbers and finding them can be challenging. It's a different grid but the challenges will be similar. Spend some time getting mentally ready. Give yourself about 90 s to prepare, before you start the next round.”			

**Figure 1:**
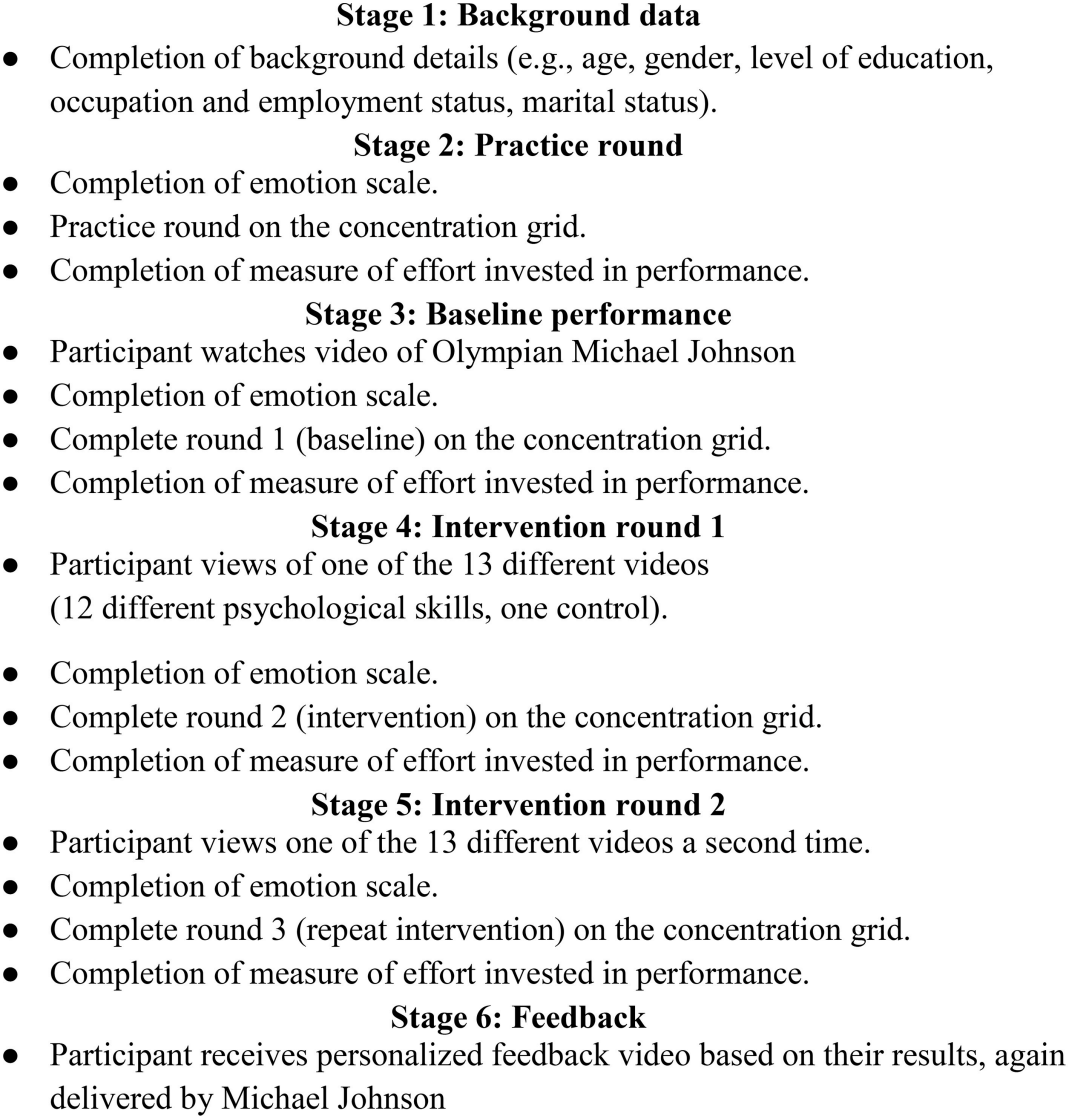
**Stages of the online protocol**.

Random allocation of participants was completed automatically by the computer program based on demographic data. Each intervention (including the control) was delivered via video clips presented by four-time Olympic gold medalist Michael Johnson. These videos guided participants through completion of self-report scales and participation in the competitive concentration grid task. Participants completed the competitive task four times: practice round, round 1 (baseline), round 2 (post intervention), and round 3 (after the intervention was repeated). The steps of the online protocol are shown in Figure 1.

### Competitive task

A competitive task was developed for the purpose of the research based on the concentration grid used in a study on emotion-performance relationships (Lane et al., 2004). This task allows capture of a large dataset via an online intervention, and has utility as a performance measure in this context. The concentration task required participants to find and click on numbers in sequence from 1 to 36 as quickly as possible from a 6 × 6 grid. Participants received a new randomly-generated grid each time.

To create perceived interpersonal competition, participants played against a computer-simulated opponent that was based on pilot data (*n* = 300). Three different opponents were generated from pilot data (slow, medium, fast), with each programmed to complete a grid in a time that was close to the participants' practice time. Participants were not told it was a computer simulated opponent.

### Measures

#### Emotion and arousal

Emotion was assessed using the items “Happy,” “Anxious,” “Dejected,” “Angry,” and “Excited” from the same-named factors in the Sport Emotion Questionnaire (SEQ; Jones et al., 2005). Jones et al. (2005) developed the five markers of emotions following extensive qualitative study of emotions experienced before competition. As multiple measures were taken and brevity was important, a simple single item was used with acknowledged limitations in terms of its relative factorial validity. A single emotion scale was computed by reverse scoring unpleasantly oriented items and adding item scores together. Each item was rated on a 7-point scale ranging from 1 (“*not at all”*) to 7 (“*extremely*”). Alpha coefficients for emotion at each completion were: Practice alpha = 0.72, Baseline alpha = 0.70, Round 1 = 0.68, and Round 2 = 0.70.

Two items “Fatigued” and “Energetic” were included to reflect arousal. Although fatigued and energetic should associate with the five emotions assessed in the SEQ, they could relate to physiological states. Energetic is an item from the Vigor scale within the Brunel Mood Scale (Terry et al., 1999), and fatigue is the factor-name from the same questionnaire. Research that has distinguished emotion from arousal demonstrates how each makes a unique contribution to performance (Karageorghis et al., 2012; Karageorghis, 2015). Each item was rated on a 7-point scale ranging from 1 (“*not at all”*) to 7 (“*extremely*”). Fatigued scores were reversed and added to Energetic scores to produce a 2-item arousal measure.

Emotion and arousal data were completed in relation to best and worst performance from memory in relation to a personal goal of their choice. Emotion and arousal data were collected prior to each round of the performance task.

#### Emotion regulation questionnaire (ERQ; Gross and John, 2003)

The ERQ is a 10-item instrument assessing individuals' habitual use of two emotion regulation strategies (reappraisal and suppression). Examples of re-appraisal include “When I want to feel more positive emotion (such as joy or amusement) I change what I'm thinking about” and “I control my emotions by changing the way I think about the situation I'm in.” Examples of suppression include “I keep my emotions to myself”; “When I am feeling negative emotions, I make sure not to express them.” Items were scored on a 7-point scale from 1 (“*strongly disagree*”), through 4 *(“neutral*”) to 7 (“*strongly agree*”). Alpha coefficients in the present study were re-appraisal alpha = 0.85, and suppression; alpha = 0.75.

#### Psychological skill usage

Psychological skills were assessed using 8-items from the Test of Performance Strategies (TOPS; Thomas et al., 1999). Examples include, “During competition I don't think about performing much,” “I rehearse my performance in my mind at competitions,” “I say things to myself to help my competitive performance”, and “I am able to relax if I get too nervous at competition.” Items were rated for frequency of use on a 5-point scale anchored by 1 (“*never”*) to 5 (“*always*”). The alpha coefficient was 0.71.

#### Performance

The computer program recorded concentration task grid completion in seconds starting from the time the task started until completion. As completion time was positively skewed with clustering for faster times and a long tail for slower ones, we corrected this lack of normality using the inverse transformation (see Box and Cox, 1964). The inverse transformation explores the rate at which participants completed the 36 number searches within the game, that is, 36/time. Accuracy was measured by assessing the number of mouse clicks. Subjective rating of performance was assessed via the item “How well did you perform?” on a 5-point scale anchored from “not at all” to “very much so.”

#### Mental effort

The Rating Scale of Mental Effort (RSME; Zijlstra, 1993) was used to assess mental effort. The RSME consisted of a scale ranging from absolutely no effort (0) to complete effort (100). Nine additional descriptive indicators on the scale (e.g., “some effort,” “extreme effort”) describe these quantities as a measure of effort.

#### Perceived influence of emotions

Two items were created to evaluate the influence of the interventions on performance and emotions. “How successfully did you manage your emotions during the game?” and “Did your emotions help your performance?” Items were rated on a 5-point scale anchored from “not at all” to “very much so.”

### Data analysis

The dataset was sufficiently large to permit complex statistical analyses and almost all analyses yield significant results due to the large sample size. This presents researchers with the challenge of how to interpret the data in the most meaningful way. Data analyses focused on examining the effects of the interventions on performance; that is, to determine whether participants performed better following the intervention, which intervention was superior, and whether following an intervention was better than practice alone. Thus, the data analysis firstly examined the effects of the interventions on task completion time. In order to address hypotheses 1 and 2, we intended to establish expected practice effects, and then investigate the extent to which interventions had any incremental effect. The second stage of data analysis examined the effects of the interventions on emotions, objective performance, subjective ratings of performance, the perceived influence of emotions on performance, and effort. Multivariate analysis of variance was used to examine the combined effect of interventions. This investigated whether improved performance associated with changes in emotions, increased effort, and more intense arousal. We examined whether motivationally focused interventions improved performance via increased arousal, contrasting results with instructional and arousal-control interventions. As the design was heavily overpowered, and as multiple tests were conducted on the same dataset, an alpha of < 0.01 was employed.

We investigated performance over time for each intervention group separately. This enabled identification of the significance and size of the effect for each group, and therefore began to unpack the effects of each intervention and control group. We also assessed differences in performance over time by group using factorial ANOVA. Delta values were calculated to account for individual differences over time.

Consistent with our first hypothesis, we expected performance to improve via practice (Stafford and Dewar, 2014). To investigate hypothesis 2, we investigated the extent to which each intervention associated with performance changes by conducting a factorial ANCOVA. This allowed identification of whether any of the psychological skills associated with larger improvements in performance than the control. To ascertain which psychological skill associated with the largest improvements in performance, delta scores were calculated and compared between the baseline round and the first intervention. Planned comparison investigated differences between psychological skills and the control group. An interrogation of *post-hoc* tests enabled analysis of whether one intervention was significantly more effective than another.

Multivariate analysis of variance (MANOVA) was used to investigate differences between interventions on a collection of variables including arousal, emotion, effort, and objective performance measures such as errors made and time to complete, along with subjective ratings of performance and beliefs about the effectiveness of the intervention in changing emotions. A multivariate approach was deemed appropriate as the intervention was proposed to have a combined effect and so if the omnibus test was significant, then univariate comparisons were investigated.

## Results

A preliminary MANOVA indicated no significant differences between groups for use of appraisal, suppression, psychological skills, emotions associated with best and worst performance, emotion experienced before completing the practice round, and perception of performance [Wilks lambda _(21, 44709)_ = 0.995, *p* = 0.754, Partial Eta^2^ = 0.000]. Therefore, groups were not significantly different on individual difference factors that could influence the uptake of psychological skills.

**Figure 2:**
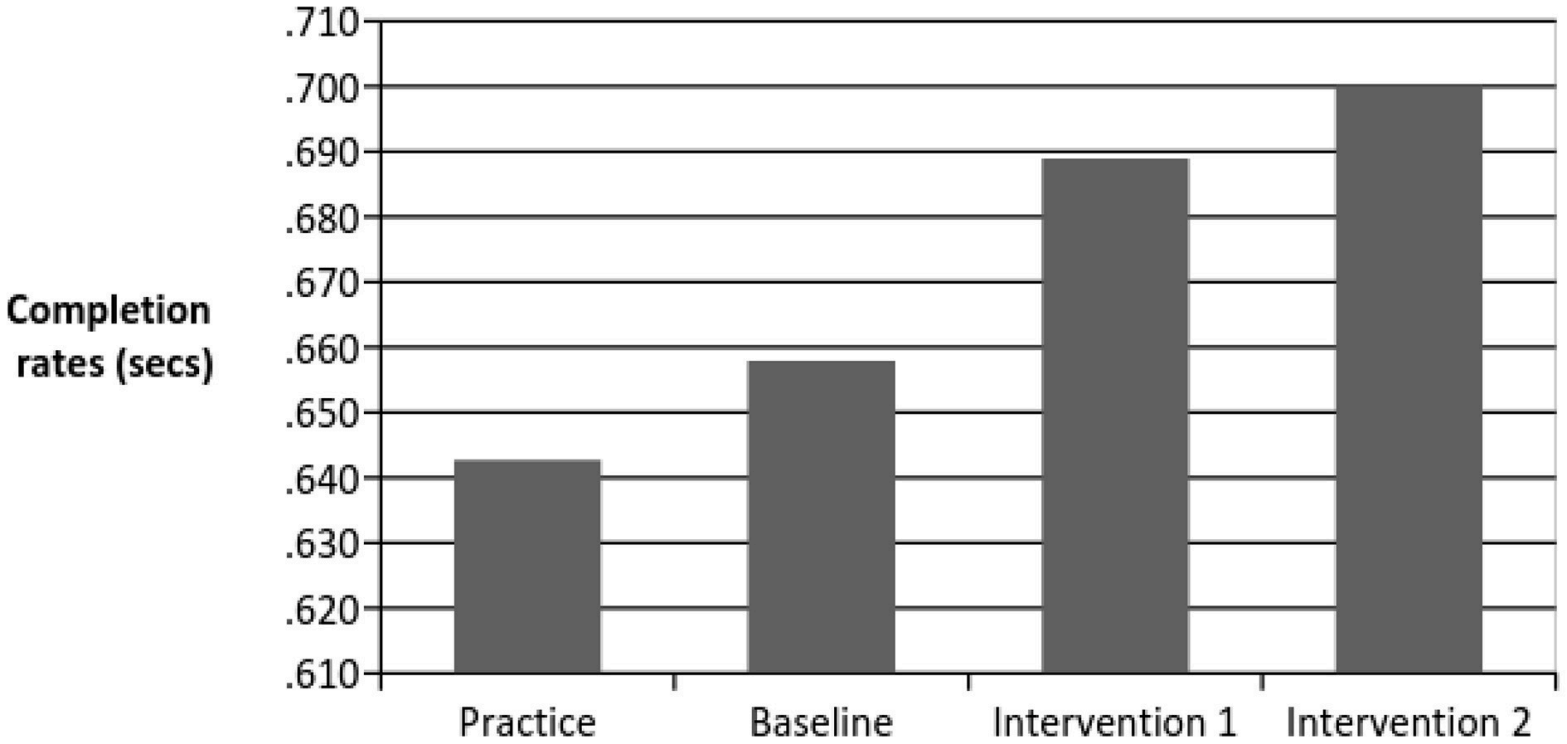
**Completion rates for performance over the course of the game**.

In terms of the effects of psychological skills on task completion time, repeated measures ANOVA results indicated that completion time improved significantly after each round [*F*_(2, 89414)_ = 2052.512, *p* < 0.001, Partial Eta^2^ = 0.04, see Figure 2]. Significant differences were found between each round [baseline and intervention; *t*_(44741)_ = 46.08, *p* < 0.001; intervention 1 and intervention 2; *t*_(44741)_ = 16.31, *p* < 0.001]. As Figure 2 illustrates, baseline times were 2.3% faster than the practice round. Times following the first intervention/control were 7.19% faster than at baseline, a figure that rose to 8.93% after completing the second intervention.

When performance was examined for each of the 12 psychological skills independently (see Table 2) results indicated that performance improved significantly in each group (*p* < 0.001), and therefore, results lend support to our first hypothesis that practice would improve performance irrespective of intervention. As Table 2 indicates, imagery and self-talk focused on outcome and process showed the largest overall effect sizes, with imagery instruction showing the weakest. Hence preliminary results indicated no significant differences between groups at baseline. Performance then improved following practice, and continued to improve following brief intervention one and two.

**Table 2:** **Effect sizes for differences in performance and emotion for each group**.

	**Performance**			
	**Overall**	**Practice vs. Baseline**	**Baseline vs. Intervention 1**	**Intervention 1 vs. Intervention 2**
If-then arousal	0.132	0.016	0.023	0.006
If-then outcome	0.189	0.014	0.066	0.003
If-then process	0.181	0.016	0.048	0.006
If-then instruction	0.146	0.014	0.028	0.013
Self-talk arousal	0.163	0.013	0.046	0.006
Self-talk outcome	0.197	0.010	0.067	0.005
Self-talk process	0.195	0.007	0.074	0.005
Self-talk instruction	0.149	0.015	0.035	0.006
Imagery arousal	0.139	0.012	0.034	0.006
Imagery outcome	0.199	0.004	0.077	0.005
Imagery process	0.200	0.013	0.066	0.006
Imagery instruction	0.104	0.015	0.017	0.006
Control	0.139	0.010	0.033	0.007

As shown in Table 3, ANCOVA results indicated a significant interaction effect between changes in performance and intervention or control group [*F*_(1, 178866)_ = 2.98, *p* < 0.001, partial Eta^2^ = 0.000], showing incremental effects for one or more of the psychological skill over another. Parameter intercepts for performance were significant for outcome-imagery, process-imagery, process-self-talk, and outcome self-talk. Therefore, in relation to our second hypothesis, results show that these three psychological skills interventions associated with faster performance than the other skills and control group (see Tables 2, 3).

**Table 3:** **Analysis of covariance analysis of changes in performance by psychological skill**.

**Parameter**	***B***	***P***	**95% Confidence Interval**		**Partial Eta Squared**
			**Lower bound**	**Upper bound**	
Intercept	0.623	0.000	0.616	0.630	0.147
Time	0.019	0.000	0.016	0.021	0.001
If-then arousal	0.009	0.084	−0.001	0.019	0.000
If-then outcome	−0.005	0.311	−0.016	0.005	0.000
If-then process	−0.001	0.834	−0.011	0.009	0.000
If-then instruction	−0.002	0.747	−0.012	0.008	0.000
Self-talk arousal	−0.004	0.482	−0.014	0.006	0.000
Self-talk outcome	−0.008	0.104	−0.018	0.002	0.000
Self-talk process	−0.005	0.372	−0.015	0.005	0.000
Self-talk instruction	0.004	0.389	−0.006	0.014	0.000
Imagery arousal	−0.001	0.773	−0.012	0.009	0.000
Imagery outcome	−0.005	0.298	−0.015	0.005	0.000
Imagery process	−0.003	0.564	−0.013	0.007	0.000
Imagery instruction	0.003	0.593	−0.007	0.013	0.000
Control	0				
If-then arousal * time	−0.001	0.525	−0.005	0.003	0.000
If-then outcome * time	0.004	0.065	0.000	0.007	0.000
If-then process * time	0.003	0.136	−0.001	0.006	0.000
If-then instruction * time	0.000	0.799	−0.003	0.004	0.000
Self-talk arousal * time	0.002	0.411	−0.002	0.005	0.000
Self-talk outcome * time	0.004	0.040	0.000	0.007	0.000
Self-talk process * time	0.004	0.028	0.000	0.008	0.000
Self-talk instruction * time	0.001	0.695	−0.003	0.004	0.000
Imagery arousal * time	−0.000	0.969	−0.004	0.004	0.000
Imagery outcome * time	0.004	0.036	0.000	0.008	0.000
Imagery process * time	0.005	0.017	0.001	0.008	0.000
Imagery instruction * time	−0.003	0.118	−0.007	0.001	0.000

Analysis of covariance analysis of changes in performance by intervention/control group.

To ascertain which psychological skill associated with the largest improvements in performance, delta scores were calculated and compared (see Table 4). An ANOVA of delta scores between baseline and the initial intervention indicated a main effect [*F*_(12, 44729)_ = 10.03, *p* < 0.001, Partial Eta^2^ = 0.003]. Planned comparison (see Table 4) indicated that outcome focused interventions delivered through each technique (if-then, imagery, and self-talk) associated with significantly faster times than the control group. Further, self-talk process and imagery-process were also associated with significantly faster times than the control group. In contrast, times for the control group were significantly faster than times for imagery-instruction. ANOVA results indicated no significant effects for technique (*p* = 0.885), focus of intervention (*p* = 0.484) and no significant interaction effect (*p* = 0.83) between round 2 and round 3. Hence, motivational interventions (process and outcome) irrespective of how they were delivered led to better improvements than control or other interventions. Arousal interventions were not significantly better than the control group. Instructional-imagery was a worse intervention than following the control group.

**Table 4:** **Differences between the intervention and control group**.

**Intervention type**	**Mean difference**	***P***	**95% Confidence interval lower bound**	**Upper bound**
If-then arousal	0.0055	0.105	−0.0011	0.0121
If-then outcome	−0.0107	0.002	−0.0174	−0.0041
If-then process	−0.0049	0.148	−0.0114	0.0017
If-then instruction	0.0037	0.268	−0.0029	0.0103
Self-talk arousal	−0.0033	0.320	−0.0099	0.0032
Self-talk outcome	−0.0111	0.001	−0.0176	−0.0046
Self-talk process	−0.0137	0.000	−0.0202	−0.0071
Self-talk instruction	0.0006	0.861	−0.0060	0.0071
Imagery arousal	0.0006	0.853	−0.0059	0.0072
Imagery outcome	−0.0144	0.000	−0.0210	−0.0078
Imagery process	−0.0110	0.001	−0.0175	−0.0044
Imagery instruction	0.0086	0.011	0.0020	0.0152

After demonstrating which interventions differed to the control in terms of time to complete the task, subsequent analysis sought to identify the collective and then specific effects on errors made, subjective performance, and emotions using a series of MANOVAs. MANOVA results indicated that at baseline there were no significant differences in pre-performance emotions and arousal, objective performance, subjective performance, and effort [Wilks lambda _(8, 44722)_ = 0.998, *p* = 0.192, Partial Eta^2^ = 0.000]. Hence, there were no significant differences at baseline.

Following the first intervention, MANOVA results indicated a significant effect [Wilks lambda _(8, 44722)_ = 0.981, *p* < 0.001, Partial Eta^2^ = 0.002], with significant effects being found for completion time, subjective performance, emotion management, perceived benefits of emotions, effort invested, emotion, and arousal (see Tables 5, 6). There was no significant effect for number of errors made and therefore instructional interventions were not any more effective than other interventions and the control. Follow-up analyses indicated that imagery arousal and if-then arousal associated with lower effort whereas self-talk for three different foci (process, instruction, and outcome) associated with significantly higher scores for effort.

**Table 5:** **Descriptive statistics for performance, effort, arousal and emotion by intervention group**.

	**Psychological skill**	**Baseline**		**Intervention**		**Intervention**	
		**M**	**SD**	**M**	**SD**	**M**	**SD**
Count of incorrect clicks	If-then arousal	0.53	1.53	0.52	1.36	0.54	1.51
	If-then outcome	0.54	1.51	0.57	1.59	0.61	1.82
	If-then process	0.53	1.37	0.54	1.84	0.56	1.86
	If-then instruction	0.56	2.28	0.54	1.96	0.55	1.67
	Self-talk arousal	0.60	2.23	0.58	1.92	0.58	1.95
	Self-talk outcome	0.60	2.29	0.64	3.30	0.63	3.86
	Self-talk process	0.61	2.80	0.61	2.97	0.63	2.36
	Self-talk instruction	0.50	1.35	0.46	1.09	0.55	1.97
	Imagery arousal	0.54	1.47	0.59	3.19	0.55	1.78
	Imagery outcome	0.56	1.93	0.66	3.40	0.64	2.22
	Imagery process	0.55	3.11	0.56	1.54	0.62	2.37
	Imagery instruction	0.57	2.72	0.63	3.56	0.69	3.65
	Control	0.59	2.55	0.64	3.36	0.63	2.94
How much effort did you put into your performance?	If-then arousal	75.38	20.03	76.11	20.32	77.41	20.66
	If-then outcome	75.99	19.14	78.56	18.97	79.55	19.51
	If-then process	76.77	18.77	78.29	19.02	79.72	19.10
	If-then instruction	76.15	19.19	78.21	18.97	79.20	19.44
	Self-talk arousal	75.80	19.57	77.40	19.36	78.69	19.57
	Self-talk outcome	76.15	19.12	79.23	18.81	80.72	18.73
	Self-talk process	75.72	19.44	79.01	18.89	80.66	18.86
	Self-talk instruction	76.45	18.93	79.18	18.69	80.02	18.95
	Imagery arousal	75.43	19.65	74.85	20.58	75.82	20.96
	Imagery outcome	75.91	19.50	78.88	19.11	79.92	19.38
	Imagery process	76.25	19.10	78.95	18.78	80.45	18.56
	Imagery instruction	76.33	18.96	78.46	18.95	79.37	19.20
	Control	75.64	19.30	77.89	19.38	79.39	19.45
How well did you perform?	If-then arousal	3.85	1.63	3.88	1.77	4.00	1.80
	If-then outcome	3.85	1.65	3.96	1.77	3.95	1.84
	If-then process	3.87	1.65	4.01	1.73	4.14	1.80
	If-then instruction	3.82	1.63	3.88	1.76	4.03	1.80
	Self-talk arousal	3.83	1.65	3.99	1.73	4.09	1.78
	Self-talk outcome	3.80	1.64	3.91	1.79	3.95	1.86
	Self-talk process	3.77	1.63	3.95	1.78	4.01	1.83
	Self-talk instruction	3.85	1.65	3.92	1.74	3.99	1.81
	Imagery arousal	3.85	1.65	3.93	1.75	3.99	1.80
	Imagery outcome	3.78	1.64	3.84	1.82	3.84	1.87
	Imagery process	3.82	1.64	3.92	1.78	3.95	1.83
	Imagery instruction	3.82	1.64	3.76	1.77	3.86	1.80
	Control	3.81	1.65	3.93	1.73	4.06	1.80
How successfully did you manage your emotions during the game?	If-then arousal	4.37	1.62	4.55	1.59	4.46	1.63
	If-then outcome	4.35	1.60	4.38	1.62	4.29	1.65
	If-then process	4.40	1.60	4.47	1.61	4.46	1.63
	If-then instruction	4.34	1.60	4.31	1.64	4.28	1.67
	Self-talk arousal	4.39	1.63	4.47	1.60	4.48	1.63
	Self-talk outcome	4.35	1.60	4.29	1.64	4.28	1.69
	Self-talk process	4.31	1.63	4.31	1.64	4.30	1.65
	Self-talk instruction	4.35	1.60	4.29	1.63	4.31	1.67
	Imagery arousal	4.38	1.63	4.53	1.62	4.41	1.68
	Imagery outcome	4.32	1.63	4.21	1.67	4.20	1.68
	Imagery process	4.36	1.59	4.30	1.63	4.25	1.67
	Imagery instruction	4.34	1.59	4.21	1.65	4.18	1.67
	Control	4.39	1.59	4.35	1.62	4.36	1.64
Did your emotions help your performance?	If-then arousal	3.30	1.74	3.27	1.78	3.26	1.81
	If-then outcome	3.32	1.74	3.34	1.75	3.24	1.78
	If-then process	3.33	1.74	3.29	1.76	3.28	1.76
	If-then instruction	3.29	1.74	3.18	1.73	3.14	1.73
	Self-talk arousal	3.26	1.72	3.32	1.78	3.32	1.79
	Self-talk outcome	3.26	1.75	3.30	1.80	3.24	1.81
	Self-talk process	3.24	1.73	3.34	1.81	3.25	1.79
	Self-talk instruction	3.28	1.75	3.23	1.75	3.19	1.77
	Imagery arousal	3.31	1.73	3.28	1.78	3.21	1.79
	Imagery outcome	3.27	1.74	3.23	1.77	3.10	1.76
	Imagery process	3.31	1.74	3.27	1.77	3.15	1.76
	Imagery instruction	3.26	1.71	3.10	1.70	3.04	1.71
	Control	3.29	1.73	3.24	1.72	3.23	1.77
Performance	If-then arousal	0.67	0.18	0.69	0.18	0.70	0.19
	If-then outcome	0.66	0.17	0.69	0.18	0.70	0.19
	If-then process	0.66	0.17	0.69	0.18	0.70	0.19
	If-then instruction	0.66	0.17	0.68	0.18	0.70	0.18
	Self-talk arousal	0.66	0.17	0.69	0.18	0.70	0.18
	Self-talk outcome	0.65	0.18	0.69	0.18	0.70	0.19
	Self-talk process	0.65	0.17	0.70	0.19	0.71	0.19
	Self-talk instruction	0.66	0.18	0.69	0.18	0.70	0.18
	Imagery arousal	0.66	0.17	0.68	0.18	0.69	0.18
	Imagery outcome	0.65	0.17	0.69	0.18	0.71	0.18
	Imagery process	0.66	0.17	0.70	0.19	0.71	0.18
	Imagery instruction	0.66	0.18	0.68	0.18	0.69	0.19
	Control	0.66	0.18	0.68	0.18	0.70	0.18
Emotion	If-then arousal	5.20	0.82	4.62	1.23	4.61	1.25
	If-then outcome	5.15	0.85	4.73	1.21	4.74	1.25
	If-then process	5.19	0.84	4.68	1.19	4.68	1.23
	If-then instruction	5.19	0.83	4.66	1.22	4.63	1.27
	Self-talk arousal	5.19	0.82	4.68	1.19	4.66	1.22
	Self-talk outcome	5.18	0.82	4.87	1.19	4.88	1.23
	Self-talk process	5.16	0.84	4.86	1.18	4.87	1.23
	Self-talk instruction	5.19	0.81	4.82	1.17	4.79	1.24
	Imagery arousal	5.16	0.84	4.57	1.18	4.54	1.21
	Imagery outcome	5.15	0.83	4.72	1.22	4.71	1.27
	Imagery process	5.17	0.82	4.76	1.21	4.73	1.26
	Imagery instruction	5.19	0.81	4.70	1.21	4.66	1.25
	Control	5.18	0.83	4.67	1.22	4.66	1.24
Arousal	If-then arousal	4.54	1.24	5.27	0.77	5.24	0.83
	If-then outcome	4.55	1.23	5.28	0.78	5.22	0.86
	If-then process	4.58	1.21	5.30	0.76	5.28	0.83
	If-then instruction	4.56	1.21	5.26	0.78	5.20	0.86
	Self-talk arousal	4.57	1.21	5.30	0.78	5.29	0.84
	Self-talk outcome	4.58	1.18	5.37	0.77	5.28	0.88
	Self-talk process	4.56	1.21	5.33	0.78	5.28	0.85
	Self-talk instruction	4.60	1.22	5.33	0.75	5.27	0.85
	Imagery arousal	4.54	1.21	5.27	0.76	5.23	0.81
	Imagery outcome	4.50	1.21	5.27	0.80	5.20	0.87
	Imagery process	4.53	1.22	5.29	0.77	5.23	0.85
	Imagery instruction	4.56	1.21	5.27	0.77	5.19	0.86
	Control	4.54	1.22	5.21	0.79	5.19	0.86

**Table 6:** **Univariate results for time, accuracy, effort, self-rated performance, emotion and arousal**.

	**Baseline**		**Intervention 1**		**Intervention 2**	
	***F***	***p***	***F***	***p***	***F***	***p***
Time to complete	1.87	0.03	4.59	0.00	3.67	0.00
Count of incorrect clicks	0.87	0.58	1.72	0.06	1.34	0.19
How much effort did you put into your performance?	1.52	0.11	15.59	0.00	17.11	0.00
How well did you perform?	1.09	0.36	4.52	0.00	7.05	0.00
How successfully did you manage your emotions during the game?	1.09	0.37	16.38	0.00	11.88	0.00
Did your emotions help your performance?	0.92	0.53	4.77	0.00	6.56	0.00
Emotion	1.32	0.20	18.97	0.00	21.04	0.00
Arousal	1.38	0.16	8.96	0.00	6.38	0.00

In terms of perceived performance, imagery instruction rated lower than other groups. Imagery instruction and imagery outcome were rated worse than control for managing emotions, whilst if-then planning arousal and imagery arousal were seen as more effective than control (all *p* < 0.01). Regarding the extent to which emotions were perceived to help performance, imagery instruction was rated lower than control, with self-talk process and if-then outcome rated higher than control. For the intensity of arousal experienced before performance, self-talk (outcome, instruction, process), and imagery process were associated with higher scores than other interventions and the control group. If-then process, self-talk process, self-talk arousal, self-talk instruction, and self-talk outcome associated with more positive emotions.

Following the second round of the intervention, MANOVA results indicated a significant effect [Wilks lambda _(8, 44722)_ = 0.981, *p* < 0.001, Partial Eta^2^ = 0.002]. Significant intervention effects for significant differences were evidenced for effort, perceived performance, beliefs about whether emotions helped performance, emotions and arousal (see Tables 5, 6). In terms of perceived performance, imagery outcome associated had the lowest rating, with if-then process, if-then arousal, and self-talk arousal rating higher than other psychological skills and the control group for managing emotion. Self-talk arousal was perceived as more helpful for performance than control.

## Discussion

The present study developed and tested the efficacy of brief online interventions on changes in performance, emotions, arousal, and effort during a competitive cognitive task. Given the number of findings resulting from data analysis, only those of statistical or practical significance are examined in the following discussion. Results indicate that performance improved significantly following each of the three psychological skills (imagery, if-then planning, self-talk) and four different foci of intervention (instructional, process, outcome, arousal control). Results also show significant improvements in performance of the control group, a finding in support of the first hypothesis concerning practice. For all 13 groups, we suggest that the rate of performance improvement is consistent with the notion that participants engaged in meaningful practice and used performance feedback to modify their approach to performance; that is, participants learned and got better at the task (see Table 5; Ericsson, 2014). Improved performance from practice is commonly reported from online gaming data, where participants are people who signed up to get better at the game, rather than to be a participant in an experiment (Stafford and Dewar, 2014). Therefore, online games represent a useful way of engaging participants in research projects. Advances in technology and the growth of users of the worldwide web suggest this approach could be an effective method of collecting large datasets and investigating worthwhile research questions.

Recent research has argued that effective online interventions tend to be ones that are attractive to users and offer feedback (Webb et al., 2010), all features of the present study. The online intervention developed in the present study contained a number of active ingredients that should have contributed to performance improvements via self-regulation (Gross and Thompson, 2007; Lane et al., 2012). Against the backdrop of knowledge that each intervention and control group associated with significant improvements in performance, our second hypothesis examined which of the 12 intervention groups was more effective, compared against the control group (Webb et al., 2010). Results revealed outcome focused goals using imagery, self-talk and if-then planning, and imagery process showed the fastest improvements over time, and moreover, were significantly more effective than the control group (see Table 3). In contrast, we did not find evidence for the proposed superiority of if-then planning.

Results indicated that imagery and self-talk focused on motivational outcome and process associated with faster performance, higher arousal, and greater effort, than participants in the control group (Tables 3, 4). Self-talk process and outcome were associated with significantly more intense pleasant emotions (see Table 5) than other interventions. Further, self-talk process was associated with positive beliefs that the resultant emotional state was functional for performance (Baumeister et al., 2007). Therefore, whilst findings show the positive effects of imagery and self-talk strategies when focused on outcome and process, it appears self-talk process had additional advantages in that participants believed it was an effective mental preparation strategy to use. Self-talk and imagery are both skills people use organically, that is without formal training (Thomas et al., 1999; Thelwell, 2015). However, it appears that self-talk is perceived to be beneficial, possibly because it is simpler to learn than imagery, which shows that some people struggle to learn imagery (Cumming and Williams, 2012). A key message from the findings of the present study is that a brief self-talk intervention focused on motivational outcomes just prior to performance intensified pleasant emotions, arousal, and effort and led to improved performance.

The importance of focus of intervention is made apparent when the same techniques (self-talk and imagery) were focused on instruction where participants performed worse than the control group. This finding emphasizes the value of identifying a relevant focus for psychological skills (Tod et al., 2011; Cumming and Williams, 2012; Devonport et al., 2016). In the present study, it appears that imagery, self-talk and if-then planning when focused on instruction was not helpful for performance. Further, imagery instruction was not perceived by participants to help regulate emotions (see Table 5), even though data on the intensity of emotions show that imagery instruction was as effective as self-talk process and self-talk outcome. However, in contrast to those interventions, imagery instruction did not increase arousal before performing. In the present study, our results suggest that in the initial stages of performing a task that requires concentration, increased arousal has beneficial effects. It is worth noting that there were no differences in the accuracy of performance between groups. Further, the psychological skills of imagery and self-talk were most effective when focused on achieving either an outcome or process focused goal; the focus of which is motivational.

The need to establish appropriate functional emotions and arousal for optimal task performance was evidenced in the present study through the relative ineffectiveness of the arousal-control focus. Arousal control interventions were associated with reduced unpleasant emotions, however, the intensity of pre-game unpleasant emotions was not overly high, thus alleviating the need to reduce their intensity (Lane et al., 2015). Previous research has found that people can believe intense unpleasant emotions as helpful for performance (Hanin, 2010; Lane et al., 2011, 2015), and so it is possible that the intervention did not match what was needed to perform better. However, participants that followed the arousal-control reported that they believed it effectively helped them regulate their emotions. An interesting expansion to the present study could have been to add on an intervention designed to increase the intensity of unpleasant emotions. Lane et al. (2015) found intervention strategies that increased arousal were associated with a significantly faster performance in the first 400 m of a 1600 m track run, suggesting an effect on decision-making. Although participants in the study by Lane et al. (2015) were not able to sustain this intensity of performance, it lends support to the notion that intense arousal can be effective for short-duration tasks. In research examining the utility of pre-task music, evidence shows that sedative music has a place in mental preparation by creating a sense of calmness and control, with stimulating music designed to increase arousal being used in relation to the demands of the task (Karageorghis et al., 2012). However, prior to performance participants prefer to select music that increases motivation.

Previous research has found that good performance on a similar concentration grid test associated with high vigor scores (Lane et al., 2004). Although Lane et al. conceptualized vigor as a mood, its components of feelings energized, active, alert and lively suggest it could also be labeled arousal. Research indicates that increased high arousal is associated with attentional narrowing (Easterbrook, 1959), that is, as arousal increases, concentration narrows and this is a characteristic that should be useful for tasks such as a concentration grid task used in the present study. In the present study, interventions that increased arousal such as self-talk process associated with the fastest performance, whereas psychological skills to reduce the intensity of emotional arousal were less helpful for performance.

Although using the arousal-control intervention in all three different skills was associated with slower performance and similar to the control group, greater support for the efficacy of this skill can be seen through analysis of its effects on perceived performance, perceived effects of the intervention, arousal, emotion, and effort. Using arousal-control interventions associated with lower scores of unpleasant emotion, strong beliefs that using the skill helped manage emotions and beliefs that performance was successful. Arousal control was effective in positively reinforcing beliefs that the skill was helpful. Interestingly, using the same variables, outcome-focused imagery was less successful. Therefore, although outcome imagery was associated with faster performance, participants did not attribute this effect to the skill. Arousal-control techniques made the connection between the desired emotional state clear to the participant. As performed improved in all groups, it should not be surprising that this served to strengthen beliefs that the intervention was effective (Hanin, 2010).

In the present study arousal-control focused psychological skills did not increase the intensity of effort invested by participants in comparison to the other interventions. Outcome and process focused skills associated with much higher scores for effort, and effort associated with faster times. The notion that increased arousal and effort might help with a short duration task requiring a narrow attentional focus makes sense when considering Easterbrook's (1959) work on attentional narrowing. However, whilst this approach to mental approach is suitable for a task that required intense effort on two short duration tasks, it might be fatiguing if the task was longer duration. Furthermore, the approach to teaching arousal-control appears effective not only in terms of regulating the intensity of emotion, but also in beliefs that it was an effective mental preparation strategy, and therefore, worth considering in future research.

We suggest that if-then planning was not an overly helpful psychological skill in the present study as its focus was on attentional changes and the short duration of the task reduced the effectiveness of such an approach. If-then planning has been found to be effective when the “if” part represents a meaningful barrier to performance (Webb and Sheeran, 2006; Achtziger et al., 2008). It is possible that when completing the task in the present study, the “if” might not have presented itself as a barrier; that is, performance was failing for other reasons, or not failing at all. Therefore, a participant was neither primed to respond with the strategy specified in the “then” aspect or the prime was not needed. If-then planning works well when individuals are faced with multiple decision-making options and make incorrect ones following reflection, such as dietary choices (Webb and Sheeran, 2006) or where there is a specific trigger to the negative response. We suggest “if-then” planning is more suitable to tasks where people make incorrect decisions, and results of the present study show that errors made was not a significant factor to explain poor performance. Future research is needed to investigate the proposed benefits of if-then planning with evidence from the present study indicating if-then arousal was more effective than control.

Although results offer evidence that one psychological skill was associated with faster times and more pleasant emotions than another, we offer some caution when transferring this finding to contexts outside of the type of performance used in the present study. Our study tested the effects of brief psychological skills on two subsequent performances. Psychological interventions in contexts such as sport typically occur over a sustained period (Gould and Maynard, 2009; Terry and Lane, 2011). Results of the present study provide evidence for the perceived benefits in terms of improved performance and functional emotions of several interventions including all four self-talk interventions (arousal, process, outcome, and instruction), and the simplicity through which these interventions could be developed and ease in which people could use them suggests investigation of their effects warrants further longitudinal research.

A key message stemming from the finding of this study is that delivering interventions online could help people perform and feel better, a finding that suggests that whether a participant was part of an intervention or control group, participation in the study associated with positive effects. Participants reported that improved performance was attributed to the effects of the intervention; perceptions that extended to the extent to which interventions helped regulate emotions. Further, at the end of the intervention, the narrator –Olympian Michael Johnson– described each individual's results and explained concepts such as emotion regulation and performing consistently. Each participant received a rich educational experience on emotion and performance management, a desirable feature of any intervention (Gaffney et al., 2013). Consistent with recent advances in online research and support (Webb et al., 2010; Brouwer et al., 2011; Cugelman et al., 2011; Kohl et al., 2013) the present study offered brief online interventions to people wishing to improve performance and recruited a sample of over 44,000 participants. Thus, although the present study benefitted from high-level mass media exposure, there are numerous potential societal benefits from teaching people effective psychological skills.

The present study has at least five limitations. First, is that the conditions in which participants took the test was unknown, and distracting experimental conditions could have contributed to the small effect sizes. Second, the quality of imagery used and whether self-talk scripts were internalized is unknown. Third, the period of training and effects were brief and whilst participants appeared to benefit, the longevity of benefits is unknown. Fourth, is that interventions could have included strategies to increase arousal. However, whilst this was considered it was deemed difficult to manage in an online setting and research that increases the intensity of unpleasant emotions warrants closer management from the research team. Fifth, whilst the effects of the selected interventions show desirable effects, this could partly be explained by the persuasive effects of receiving the intervention from Michael Johnson, an Olympic Champion who is both a credible person and known advocate of mental training. An interesting comparison would be to investigate the effects of the intervention when delivered by a less-esteemed source.

In conclusion, the present study represents a large-scale intervention study that makes use of an online method to gather and test a large dataset pertaining to competitive game performance. Results suggest that psychological skills focused on outcome and process goals had the strongest positive effects on performance speed, coupled with increased positive emotions. The present study offers a method that could supplement the methods by which individuals organically learn psychological skills. Future research should invest in technology to develop interesting and engaging interventions to teach, and investigate the efficacy of, psychological skills.

## Author contributions

All authors (AL, PT, IM, TD, AF, CB, DS, and AN) contributed to this work. AL, PT, IM, AF, CB, DS were involved in the conceptual design of the project whilst AL, TD, AF, and AN were involved in the analysis of data. All authors participated in drafting and revising the current manuscript. All authors provided final approval of the manuscript and each has agreed to be accountable for the integrity of the work.

### Conflict of interest statement

The authors declare that the research was conducted in the absence of any commercial or financial relationships that could be construed as a potential conflict of interest.
